# Sexual activities during the COVID-19 pandemic in Indonesia

**DOI:** 10.1186/s12301-021-00227-w

**Published:** 2021-08-16

**Authors:** A. H. Wisda Kusuma, Sakti R. Brodjonegoro, Indrawarman Soerohardjo, Ahmad Z. Hendri, Prahara Yuri

**Affiliations:** Urology Division, Department of Surgery, Faculty of Medicine, Public Health, and Nursing, Universitas Gadjah Mada / Dr. Sardjito Hospital, Jl. Kesehatan No.1, Yogyakarta, 55281 Indonesia

**Keywords:** COVID-19, Pandemic, Sexual activity, Depression, Fear, Anxiety, Isolation

## Abstract

**Background:**

Corona Virus Disease 2019 (COVID-19) has spread globally starting from late 2019. The WHO declared it a global pandemic in March 2020, causing nations around the world to introduce various control measures to halt the rapid spread of the disease, such as quarantines, lockdowns, and work from home (WFH) policies. These policies often force people to spend more time at home with their cohabitants, or possibly sexual partners. Various negative feelings experienced during those policies are considered to affect the general mood and sexual life of the population. This study aimed to investigate the difference in mood and sexual activity before and during the COVID-19 pandemic.

**Methods:**

This research was a cross-sectional pilot study. Authors collected data from 131 randomly selected, sexually active volunteer subjects using a self-administered online questionnaire. Subjects’ mood status, behavior, and frequency of sexual intercourse before and during COVID-19 pandemic in Indonesia were analyzed.

**Results:**

Subjects consisted of 67 (51.1%) men and 64 (48.9%) women. Our analysis shows that there was a decline in overall mood scale, and also sexual activity frequency, before and during the pandemic (4.63 vs. 4.03; 80.2% vs. 67.9%, respectively). The COVID-19 pandemic control measures may enable subjects to have more time with their sexual partners at home, but it does not increase the frequency of their sexual activities.

**Conclusion:**

There was a slight decrease in overall mood scale and sexual activity frequency during the COVID-19 pandemic recorded among subjects. The authors suspect that depression symptoms, fear, anxiety, irritability, boredom, confusion, and feeling of being isolated experienced during strict pandemic control measures, caused by stressors such as job loss, decreased monthly income, and the current state of the pandemic are influencing these phenomena.

## Background

In December 2019, Chinese health authorities reported a cluster of pneumonia cases of unknown etiology in Wuhan City, Hubei province, People’s Republic of China. In January 2020, the Chinese Center for Disease Control (CDC) identified a novel virus named *novel coronavirus 2* (nCoV-2) as the causative agent for these cases, and confirmed human-to-human transmission of the virus [1].

The *Severe Acute Respiratory Syndrome novel Corona Virus-2* (SARS-nCoV-2) that causes Corona Virus Disease 2019 (COVID-19) has spread rapidly since then, causing the World Health Organization (WHO) to declare the disease a global pandemic on March 11, 2020 [1].

The main route of transmission for the new coronavirus is through direct transmission, including coughing, sneezing, and inhalation of droplets, and contact with oral, nasal, or eye mucous membranes. Current evidence also shows fecal–oral transmission. Although SARS-nCoV-2 has been found in the sperm of 34 Chinese men recovering from mild COVID-19, sexual transmission has not been documented yet. However, the nature of sexual activities, which require being inside someone’s personal space, may ease the transmission of the virus via its main routes of transmission [1].

The COVID-19 pandemic has caused disruptions to health care and social systems around the world [2]. Strict control measures made by governments such as quarantines, lockdowns and work from home (WFH) policies often severely limit people’s freedom to travel and perform their daily activities. In some cases, the policies force people to live alone, or side by side with their cohabitants, or possibly sexual partner for 24 h a day in a limited space. The sudden change in the way of living may result in increased stress, anxiety, and other negative emotions, which in turn, gives negative impacts on their overall mood and sexual satisfaction [3].

Large-scale disaster events such as the 2008 earthquake in Wenchuan, China are known to cause decreased sexual frequency, sexual satisfaction, and desire for pregnancy. Liu et al. [4] stated that 67.1% of female earthquake victims studied would terminate their pregnancy if they became pregnant. Likewise, the negative impact of the COVID-19 pandemic on sexual and reproductive health has been reported by many studies around the world. Studies in China and Turkey have shown that the COVID-19 pandemic has led to decreased sexual satisfaction in both men and women [5, 6].

A satisfying sexual life may improve a person’s general well-being and quality of life. Studies have correlated a satisfactory sexual life with increased satisfaction in one’s health, increased level of trust and intimacy with a partner, and less use of immature psychological defense mechanisms [3]. Women with a satisfying sexual life have fewer cardiovascular events in a 5 year period. One similar study in men showed an inversed correlation between the frequency of sexual activity and cardiovascular events [7]. Furthermore, individuals with more frequent sexual activities may have a better and more efficient immune system. A study of 112 college students showed a significantly higher salivary Immunoglobulin A level in students who had sex more than once or twice per week [7]. Successful sexual activities may also improve mood, sleep quality, relaxation, reduce stress and anxiety, which contribute to the prevention of post-traumatic stress and anxiety disorders [8]. By understanding the benefits of having a satisfying sexual life, scholars around the world have given recommendations on safe sexual activity during the COVID-19 pandemic when even survival is a challenge [2, 8].

The authors aimed to investigate the change in mood and sexual activity frequency among male and female subjects in Indonesia during the COVID-19 pandemic, with the hope of gaining better attention, and promoting better interventions to sexual and reproductive health changes during this challenging time in Indonesia.

## Methods

This research was a cross-sectional pilot study. The authors used random consecutive sampling methods and conducted a survey with an online self-administered questionnaire using *Google Form*® as the platform. The sampling period lasted for 1 month between November 2020 and December 2020. There were 131 sexually active subjects who completed the questionnaire from 11 major provinces in Indonesia. The questionnaire required subjects to give personal information such as their mood status, sexual behavior, and sexual activity frequency before and during the COVID-19 pandemic. In addition, subjects were also required to give their subjective views on how the pandemic affects their sexual life, and their views on the ideal sexual activity frequency. Subjects were explained about the aims and data confidentiality of the study before completing the questionnaire.

Mood status was measured using the *Depression Intensity Scale Circles* (DISCs). The DISC score is quantified using a scale of 1–6 in the form of research results. A scale of 1 indicates the subject is *very depressed*, whereas a scale of 6 indicates the subject is *very not depressed* (happy) [9].

In the event that a potential subject disagreed to complete the survey of the study, the individual could simply not finish filling out the questionnaire without any consequences.

This study obtained approval from the Medical and Health Research Ethics Committee of the Faculty of Medicine, Nursing, and Public Health Universitas Gadjah Mada with reference no KE/FK/1239/EC/2020. There are no conflicts of interest in this study. This study was funded by the authors, without any involvements of sponsors.

## Results

There were 131 sexually active subjects who participated in this study, and the subjects’ characteristics are shown in Table 1.

**Table 1:** Subjects’ characteristics

Gender		
Male	67	(51.1%)
Female	64	(48.9%)
Ages		
Range (mean)	22–56	(28.74)
Province		
Jakarta	21	16.03%
West Java	18	13.74%
Central Java	18	13.74%
Yogyakarta	32	24.43%
East Java	10	7.63%
Bali	10	7.63%
Sumatera	7	5.34%
Borneo	5	3.82%
Celebes	5	3.82%
Banten	4	3.05%
Papua	1	0.76%
Occupation		
Unemployed	13	9.92%
Student	21	16.03%
Professional	48	36.64%
Trader/businessman	12	9.16%
Officer	32	24.43%
Farmer/labor	2	1.53%
Soldier/police	3	2.29%
Education		
High school	7	5.34%
Graduate	113	86.26%
Post graduate	11	8.40%
Marital status		
Married	95	71.97%
Unmarried	37	28.03%
Sexual orientation		
Heterosexual	126	96.18%
Homosexual	3	2.29%
Bisexual	2	1.53%
Chronic illness		
Erectile dysfunction	2	1.53%
Asthma	1	0.76%
Psychiatric disorder (depression)	1	0.76%
No chronic illness	127	96.95%
Use of PDE-5i		
Yes	4	3.05
No	127	96.95
Sexual partner		
Wife/husband	95	72.51
Boy/girlfriend	31	23.66
Secret lover	2	1.52
Friend	3	2.29
Ways to have sex		
Penis-vagina only	88	67.2%
Penis-vagina + oral sex	36	27.5%
Oral sex only	1	0.8%
Penis-vagina + oral sex + anal sex	2	1.5%
Oral + Anal sex	2	1.5%
Anal sex	1	0.8%
Masturbate	1	0.8%

Responses showed that from the 131 samples, consisting of 67 males and 64 females, the province with highest number of subjects was Yogyakarta (24.42%), and Papua was the province with the least number of subjects (0.76%). Most of the subjects’ occupations were professionals (36.64%), with farmers/laborers (1.53%) being the fewest jobs. Most of the subjects were undergraduates (86.24%), married (71.97%), and are heterosexual (96.18%). There are several confounding factors, namely a history of erectile dysfunction, asthma, and a documented psychiatric problem. Among the subjects there was also a history of using PDE5-inhibitors (3.05%). The majority of sexual partners involved their wives/husbands (72.51%), and mostly penis-vagina penetration (67.2%).

Frequency and behaviors of sexual activities are recorded as listed in Table 2.

**Table 2:** Subjects’ sexual behaviors

Criteria	Before pandemic (July–December 2019)		During pandemic (January–June 2020)	
	*n*	%	*n*	%
Routine sexual activities				
Yes	105	80.2	89	67.9
No	26	19.8	42	32.1
Frequencies				
< 1× a week	33	25.4	44	24.1
1× a week	27	20.4	25	19.4
2× a week	31	23.8	26	20.2
3× a week	32	24.6	29	22.5
Daily	6	4.6	4	3.1
> 1× a day	1	0.8	1	0.8
Mood scale (mean)	4.63		4.03	
Condom use				
Always	14	10.8	14	10.8
Often	8	6.2	10	7.7
Seldom	31	28.5	31	28.5
Never	71	54.6	75	57.7

Results showed that there was a decrease in sexual activity during the pandemic (67.9%), compared to before the pandemic (80.2%), along with a decrease in the subject's mood status. Most subjects have sexual activity with a frequency of < 1× per week. There was no significant difference in condom use between before and after the pandemic.

Among 131 survey participants, there were several subjective questions asked about sexual activity during the COVID-19 pandemic, as listed in Table 3.

**Table 3:** Subjects’ responses about sexual activities changes

No	Question	Answer	*n*	Percentage
1	Does COVID-19 pandemic affect your sexual activity?	Yes	70	53.8%
		No	60	46.2%
2	Do you feel you have more time for sexual activity?	Yes	41	33.3%
		No	23	18.7%
		Same	59	48%
3	Does this COVID-19 pandemic encourage you to engage in more sexual activities?	Yes	46	35.1%
		No	85	64.9%
4	In your opinion, when there was no COVID 19 pandemic, how often should sexual relations be done?	< 1× per week	9	7
		1× per week	19	14.7
		2× per week	40	31
		3× per week	48	37.2
		Daily	12	9.3
		> 1× per day	1	0.8

Subjectively, it was seen that 53.8% of respondents admitted that the COVID-19 pandemic affected their sexual activity, although most (48%) said that there was no difference in free time for sexual activity before and during the pandemic. A total of 64.9% of the subjects also admitted that the COVID-19 pandemic did not encourage them to be more passionate about sexual activities. According to 37.2% of the subjects, the ideal frequency of sexual intercourse is 3 times per week.

## Discussion

The COVID-19 global pandemic has caused disruptions to healthcare system, deterioration of social life quality, reduction in income, and a high number of deaths around the world [2, 3, 5, 10]. This study aimed to investigate the effects of COVID-19 pandemic and the various governmental policies issued against its transmission on overall mood, and sexual activity frequency.

Hamilton et al. [11] reported that chronic stress at a high level may contribute to lower level of genital sexual arousal. The decrease in genital sexual arousal is mainly due to psychological factors (i.e., distraction) and hormonal factors (i.e., increased cortisol level) [11]. Similarly, Liu et al. [4] found a decrease in the frequency of sexual intercourse after the 2008 Wenchuan earthquake in China. In contrast, Hall et al. [12] investigated the influence of stressful lifestyles on sexual behavior, and reported significantly more frequent sexual activity among women in highly stressful times compared with less stressful times. The study analyzed the proportion of sexually active weeks among female subjects, and reported that the proportion of sexually active weeks for women with stress and depression were 43% and 40%, respectively. These responses were significantly higher compared to the lower proportion of women without stress and depression (35% and 35%, respectively) (*P* < 0.001) [12].

Our results show that there was a slight decrease in subjects’ sexual activities during the COVID-19 pandemic. Results show declining trends in overall frequency of sexual activities (80.2% vs. 67.9%) and mood scale (4.63 vs. 4.03) during the pandemic.

The WHO defines mental health as “a state of complete physical, mental, and social well-being.” By that definition, it is clear that mental health is not merely the absence of any mental disorders [3]. Several studies in 2020 showed individuals who underwent quarantine during the COVID-19 pandemic reported depression symptoms, fear, anxiety, irritability, boredom, confusion, and feelings of being isolated [3, 13]. Duration of the quarantine also plays a role in the post-quarantine psychological health. Individuals quarantined for more than 10 days have a higher chance of developing post-traumatic stress symptoms than those quarantined for less than 10 days. Socioeconomic factors are most likely to be the causative factors for these symptoms. People have reported fear of infecting others, frustration and boredom, inadequate information, insufficient quarantine supply, and financial issues were the major stressors during their quarantine period in the COVID-19 pandemic [13].

Sexual activity is strongly linked with both mental physical health [3]. Decreased overall mood and mental health disorders such as depression symptoms and anxiety have been shown to negatively impact sexual desire, arousal, frequency, and overall sexual satisfaction in both genders [6, 12, 14]. A German study showed that among 4955 subjects, most respondents with a self-reported disease (physical and/or mental) stated that it was associated with a marked impairment in their sexual life, regardless of whether the diseases have any clear relation to sexuality [14].

In Spain, most subjects of a large-scale study stated that the main reason for them to have sexual intercourse is to look for emotional intimacy, and to satisfy their need to love and be loved. In struggling times such as a global pandemic, the comfort of sexual activity may offer people a much-needed help in reducing post-traumatic stress and anxiety disorders [8]. During early COVID-19 responses in the United States of America (USA), better mental health outcomes were documented among those who maintained frequent in-person social and sexual relationships, while virtual remote relationships, however, did not improve mental health outcomes [15].

Given the clear benefits of having a satisfactory sexual life, it is very important to enable people to live their sexual life satisfyingly, even in this time of struggling for safety and survival. Sexual activities, however, must not increase the spread of COVID-19, which may prolong the pandemic, and further increase the mental and sexual health strain of the people.

Experts have given their recommendations on safe sexual intercourse during the COVID-19 pandemic. The one rule considered to be safest is that “you are your safest sexual partner,” therefore, encouraging masturbation as a method to achieve sexual satisfaction without the risk of contracting COVID-19 [8, 16]. It is important to remember that while the COVID-19 virus does not spread through sexual intercourse, the nature of sexual intercourse requires people to be in touch with each other, therefore not maintaining safe distance to reduce the risk of contracting COVID-19 [1]. Cabello et al. [8] recommended that kissing and oral sex should be avoided. Proper hand washing is needed to avoid viral transmission by touching the T-zone (mouth, nose, and eyes) during sexual intercourse. Sexual intercourse with a partner suspected of and/or confirmed with COVID-19 should be avoided until the individual has completed the necessary management protocols. The same caution should be applied for a partner in contact with COVID-19 patients such as doctors and nurses. When starting a sexual relationship with a new partner, it is advised to wait until a 10-day incubation time passes without symptoms before starting to engage in sexual intercourse [8].

## Conclusions

Our study provides preliminary data on the effects of the COVID-19 pandemic on overall mood and subjects’ sexual life. The authors found that the COVID-19 pandemic has caused decreased overall mood, and slightly lower sexual activity frequency among subjects. Feelings experienced during the pandemic such as depression symptoms, fear, anxiety, irritability, boredom, confusion, and feeling of being isolated might play a role in the decreasing of overall mood and sexual desire, which results in less frequent sexual activities.

This study should not be interpreted as evidence in support of loosening the necessary restrictions in place to prevent COVID-19 transmission. Subjects’ responses indicate that the impact of an unsatisfactory sexual life during the COVID-19 pandemic needs to be addressed in the current COVID-19 response policies.

This research was a pilot study, therefore, the results can only present preliminary data concerning this important topic, particularly in Indonesia. The authors strongly recommend conducting a larger, multi-centered study to better describe the effects of COVID-19 pandemic on overall mood and sexual activity frequency.

## Data Availability

The data that support the findings of this study are available from the corresponding author, AHW, upon reasonable request.

## Abbreviations

CDC: Centers for disease control and prevention
COVID-19: Corona Virus Disease-2019
DISCs: Depression intensity scale circles
HIV: Human immunodeficiency virus
WFH: Work from home
